# New Polycyclic Red Luminescent Compounds Based on Carbonyl/Nitrogen Skeleton for Efficient Narrow-Spectrum OLEDs

**DOI:** 10.3390/ma18174000

**Published:** 2025-08-26

**Authors:** Zhiwei Wu, Peng Zou, Ziwei Chen, Ben Zhong Tang, Zujin Zhao

**Affiliations:** 1 State Key Laboratory of Luminescent Materials and Devices, Guangdong Provincial Key Laboratory of Luminescence from Molecular Aggregates, South China University of Technology, Guangzhou 510640, Chinaziweichen980515@163.com (Z.C.); 2 Guangdong Basic Research Center of Excellence for Aggregate Science, School of Science and Engineering, The Chinese University of Hong Kong, Shenzhen 518172, China

**Keywords:** organic light-emitting diodes, electroluminescence, carbonyl/nitrogen skeleton, hybridized local and charge-transfer state, narrow emission spectrum

## Abstract

Advances in OLED display technology have increased the demand for high-performance luminescent materials, yet efficient red emitters with narrow emission spectra remain rare. Here, two new polycyclic compounds (O-QA and S-QA) are designed by incorporating oxygen/sulfur into a carbonyl/nitrogen skeleton. Photophysical and theoretical studies reveal their hybridized local and charge-transfer state characteristics. In toluene, O-QA and S-QA show photoluminescence peaks at 586/579 nm with narrow emission spectra, while doped films exhibit strong red emissions peaking at 598/600 nm with high PL quantum yields of 67%/60%. The OLEDs using these emitters achieve red electroluminescence (EL) peaks at 598/602 nm, and attain maximum external quantum efficiencies of 7.36%/14.54%. This work may provide guidance for the development of narrow-spectrum red emitters based on carbonyl/nitrogen frameworks.

## 1. Introduction

Organic light-emitting diodes (OLEDs) have demonstrated remarkable progress in recent years, emerging as a highly promising technology for next-generation display and lighting [1,2]. The development of efficient and high color purity organic light-emitting materials is essential for the preparation of OLEDs for wide color gamut high-definition displays [3,4]. According to quantum spin statistics, the combination of holes and electrons in OLEDs normally forms 25% of the singlet excitons and 75% of the triplet excitons [5]. For most fluorescent materials, only singlet excitons can be harvested, which leads to low efficiencies of the devices. In recent years, researchers have devoted great efforts to enhance triplet exciton utilization of purely organic materials, and a variety of purely organic luminescent materials capable of utilizing triplet excitons via different photophysical mechanisms, including triplet–triplet annihilation (TTA) [6], hybridized local and charge-transfer (HLCT) [7,8], thermally activated delayed fluorescence (TADF) [9,10,11,12,13], room-temperature phosphorescence [14], and radical-based doublet emission [15,16]. The HLCT emitters represent an advanced class of organic luminescent materials that combine the advantages of locally excited (LE) and charge-transfer (CT) states. In this situation, the higher triplet state (T_2_ or T_n_) can be transformed into the lowest singlet state (S_1_) by a high-lying reverse intersystem crossover (hRISC), which effectively improves the exciton collection capacity.

The full width at half maximum (FWHM) of the emission spectrum is one of the important influencing factors in OLED materials. A large full width at half maximum of the emission spectrum of luminescent compound may limit their use in high-definition displays, while narrow-spectrum luminescent materials are favored for achieving high color purity. In 2016, Hatakeyama et al. reported intriguing polycyclic aromatic materials embedded with boron atom and nitrogen atom [17], which have gained widespread attention owing to their unique multiple resonance (MR) property capable of realizing extremely narrow FWHM and excellent optoelectronic performance at the same time [18]. However, the syntheses of MR materials based on boron/nitrogen skeleton are quite complicated with low yields, which are not favorable for mass preparation. Recently, carbonyl/nitrogen skeletons are emerging as another option for developing narrow-spectrum materials [19,20]. Due to the short-range charge-transfer feature, the emissions of most carbonyl/nitrogen-based MR materials are currently located in the blue-green region, while those with emissions in the red region are relatively few [21,22].

**Figure 1:**
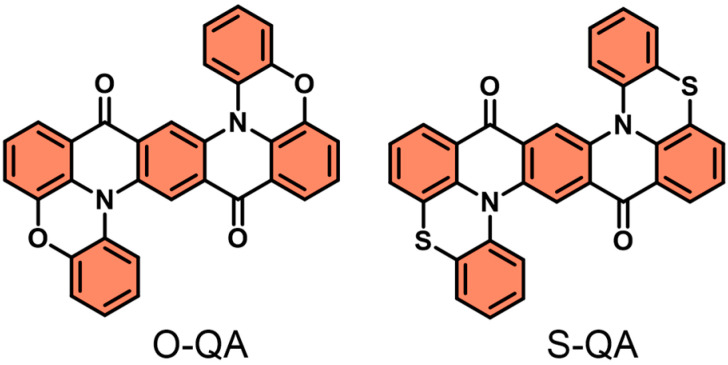
Molecular structures for O-QA and S-QA.

In this work, we design and synthesize two new polycyclic luminescent compounds (O-QA and S-QA) based on carbonyl/nitrogen skeleton [23,24,25], which are induced to red-shifted emissions by increasing intramolecular charge-transfer effect by means of embedding S or O atoms (Figure 1). They have high molecular rigidity, which is conducive to achieving narrow emission spectra. O-QA and S-QA show strong photoluminescence (PL) emissions with peaks at 586 and 579 nm and narrow FWHMs of 48 and 64 nm in toluene solutions, respectively, and provide efficient red EL emissions in doped OLEDs with EL peaks at 598 and 602 nm, and good maximum external quantum efficiencies (EQE_max_s) of 7.36% and 14.54%, respectively.

## 2. Results and Discussion

### 2.1. Synthesis and Characterization

**Scheme 1:**
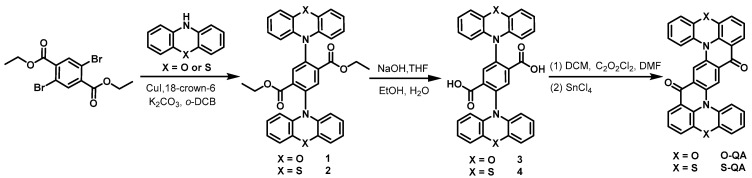
Synthetic routes of O-QA and S-QA.

The target compounds (O-QA and S-QA) are synthesized by Ullmann coupling, hydrolysis, and Friedel–Crafts acylation reactions, and the synthetic routes are shown in Scheme 1. The structures of the intermediates and the final products are fully characterized by NMR (Figures S1–S6) and high-resolution mass spectrometry. The thermal properties of O-QA and S-QA are evaluated by thermogravimetric analysis (TGA) and differential scanning calorimetry (DSC). TGA shows that the decomposition temperature (*T*_d_, 5% initial weight loss) is 444 °C for both O-QA and S-QA (Figure S7a), implying the excellent thermal stability, which is beneficial for preparing vacuum-deposited OLED devices. The glass-transition temperatures are not found in DSC curves in the range of 100–300 °C (Figure S7b). Their electrochemical behaviors in dichloromethane are investigated by cyclic voltammetry (CV) (Figure S7c). O-QA and S-QA exhibit similar oxidation and reduction potentials, with highest occupied molecular orbital (HOMO) energy levels of −5.33 and −5.40 eV, respectively, and lowest unoccupied molecular orbital (LUMO) energy levels of −3.22 and −3.23 eV, respectively.

### 2.2. Theoretical Calculation

**Figure 2:**
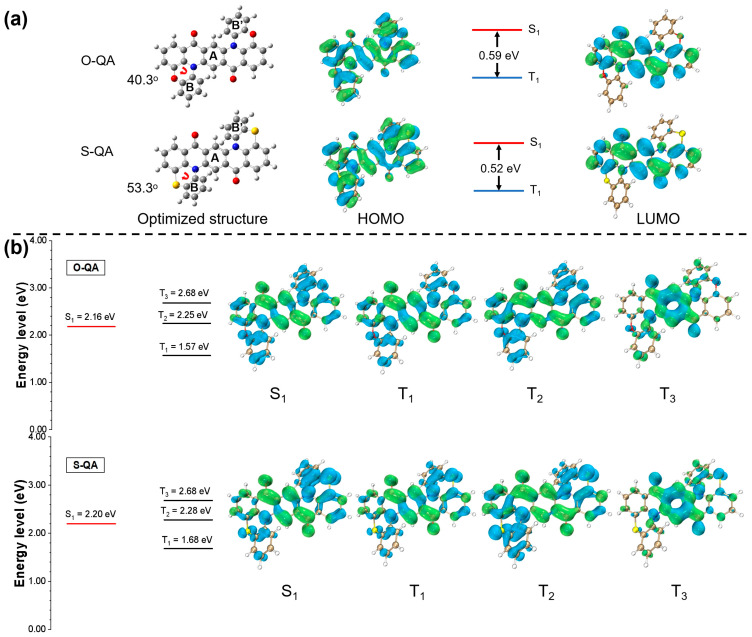
(**a**) Optimized geometries and frontier orbital distribution of S_0_ state. (**b**) Calculated energy levels of singlet and triplet states and analysis of hole (blue) and electron (green) distributions.

The geometrical and electronic structures of O-QA and S-QA are simulated by theoretical calculations (Figure 2a). From the optimized ground-state geometries, it is observed that both compounds exhibit distorted molecular configurations. S-QA has a larger dihedral angle of 53.3° between the central benzene ring (A ring) and the peripheral tilted benzene ring (B or B′ ring) relative to the dihedral angle of 40.3° for O-QA, probably due to the larger radius of S atom than O atom. In addition, the structural changes between ground (S_0_) and S_1_ states are further analyzed using root mean of squared displacement (RMSD). The results show that the conformational change of S-QA is greater than O-QA (Figure S8), and thus it is expected that the S-QA has broader emission spectrum than O-QA. Their HOMOs are delocalized throughout the entire molecular skeletons, whereas the LUMOs are mainly concentrated in the central parts, suggestive of the HLCT feature of both compounds.

For HLCT materials, their LE components provide high radiative transition rates and the CT components not only regulate the emission wavelengths but also open up hRISC processes, thus realizing full utilization of the triplet excitons [26,27]. The energy levels and electron-hole distributions of the excited states are calculated as well. As shown in Figure 2b, it is found that the lowest excited singlet (S_1_), lowest excited triplet (T_1_) and second triplet excited (T_2_) states have a mixture feature of LE and CT components, further confirming the presence of HLCT characteristics [28]. The natural transition orbit (NTO) calculation reveals that the HONTOs in S_1_, T_1_, and T_2_ states all have HLCT characteristics (Figure S9). The energy splitting (Δ*E*_ST_) values between the S_1_ and T_1_ states are calculated as 0.59 and 0.52 eV for O-QA and S-QA, respectively. Such large Δ*E*_ST_ values indicate that it is difficult for them to have TADF properties. The energy gaps between T_1_ and T_2_ states of O-QA and S-QA are 0.68 and 0.60 eV, respectively, and such large energy difference is unfavorable for internal conversion from T_2_ to T_1_ states. Meanwhile, the energy level of T_2_ state lies close to that of S_1_ state, indicating hRISC from T_2_ to S_1_ states may occur, which is beneficial for the utilization of triplet excitons [29,30,31].

### 2.3. Photophysical Properties

**Figure 3:**
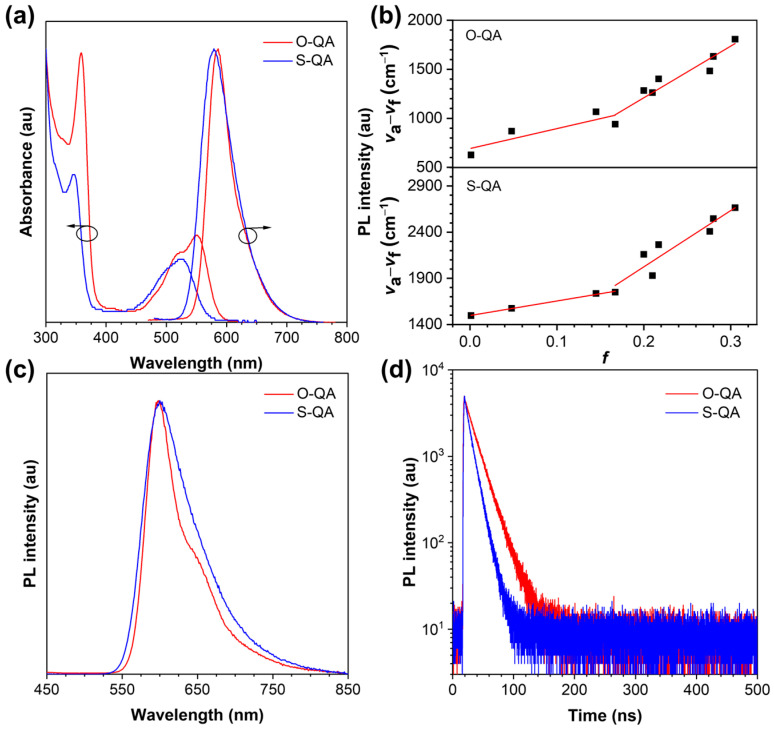
(**a**) Absorption and PL spectra in dilute toluene solutions (10^−5^ M) and (**b**) solvatochromic Lippert–Mataga plots of O-QA and S-QA. (**c**) PL spectra and (**d**) transient PL decay curves of O-QA and S-QA in doped films.

The photophysical properties of both compounds in solution and doped film states are studied. The UV-vis absorption spectra of both compounds in toluene solutions have strong absorption bands below 400 nm from π-π* transitions (Figure 3a). The absorption maxima of O-QA and S-QA are located at 550 and 524 nm (Table 1), respectively, which are associated with CT states. O-QA and S-QA in toluene solutions show orange-red PL emissions with peaks at 586 and 579 nm, respectively. O-QA has a narrower FWHM of 48 nm than S-QA (64 nm) because of the more planar and rigid structure of O-QA [32]. On the other hand, the solvatochromic effects of O-QA and S-QA are studied in organic solvents with different polarity. As shown in Figure S10, the PL peaks of O-QA and S-QA are red-shifted along with the increase of solvent polarity, and the HLCT characteristics of O-QA and S-QA are confirmed by a two-section linear relationship between Stokes shift (*v*_a_ − *v*_f_) and solvent orientational polarizability *f* (ε, *n*), as fitted by the Lippert–Mataga model [33,34] (Figure 3b, Figure S10 and Tables S1 and S2). Meanwhile, both compounds exhibit single-exponential PL decay in toluene solutions (Figure S11), which is in good agreement with the HLCT feature. The absolute PL quantum yields (*Φ*_PL_s) of O-QA and S-QA in toluene solutions are 55% and 49%, respectively.

The doped films of O-QA and S-QA in the host of 9-(3-(9*H*-carbazol-9-yl)phenyl)-9*H*-3,9′-bicarbazole (*m*CPBC) (doping concentrations are 1 wt% for O-QA and 5 wt% for S-QA) show PL peaks at 598 and 600 nm, and FWHMs of 55 and 79 nm, respectively (Figure 3c). There are slight redshifts and broadening in PL spectra compared to those in toluene solutions, due to intermolecular interactions in the aggregated state. Their transient PL decay spectra in doped films show single-exponential decay with fitted lifetimes of 18.6 and 11.1 ns for O-QA and S-QA, respectively (Figure 3d), and no delay component are observed. Based on the fluorescence and phosphorescence spectra of the doped film at 77 K (Figure S12), the Δ*E*_ST_ values of O-QA and S-QA are estimated to be 0.29 and 0.26 eV, respectively. The large Δ*E*_ST_ values indicate that the TADF phenomenon could be difficult to occur. The *Φ*_PL_s of O-QA and S-QA in doped films are 67% and 60% respectively, and the radiative transition rates (*k*_r_) are 3.60 × 10^7^ and 5.41 × 10^7^ s^−1^, respectively.

### 2.4. Electroluminescence Behaviors

**Figure 4:**
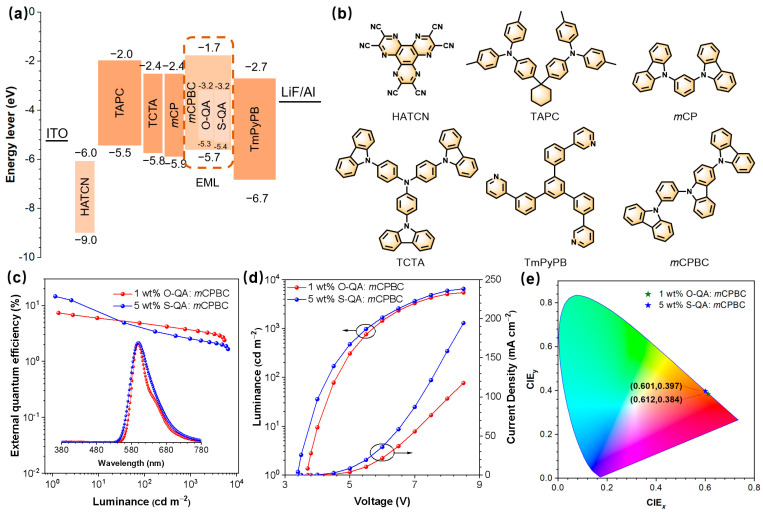
(**a**) Device configurations, (**b**) structures of functional layers, (**c**) external quantum efficiency–luminance plots (inset: EL spectra), (**d**) luminance–voltage–current density plots, and (**e**) CIE coordinates of doped devices of O-QA and S-QA.

Considering the favorable photophysical properties and good thermal stability of these two compounds, their EL behaviors are further evaluated by fabricating doped devices with the structures of ITO/HATCN (5 nm)/TAPC (50 nm)/TCTA (5 nm)/*m*CP (5 nm)/EML (20 nm)/TmPyPB (70 nm)/LiF (1 nm)/Al. The doped films of these compounds with the doping concentrations of 1, 3, 5, and 10 wt% in *m*CPBC host function as emitting layers (EMLs), 1,4,5,8,9,11-hexaazatriphenylenehexacarbonitrile (HATCN) and lithium fluoride (LiF) serve as hole-injection layers and electron-injection layers, respectively, 1,1-bis[(di-4-tolylamino)phenyl]cyclohexane (TAPC) and tris(4-carbazoyl-9-ylphenyl)amine (TCTA) function as hole-transporting layers, 1,3-bis(carbazol-9-yl)benzene (*m*CP) works as exciton-blocking layer and electron-blocking layer, and 1,3,5-tri(m-pyrid-3-yl-phenyl)benzene (TmPyPB) performs as electron-transporting layer. The EL performances of all the devices are summarized in Figure 4, Figure S13, and Table 2. O-QA and S-QA show the best EL results at 1 wt% and 5 wt% doping concentrations, respectively, which display narrow-spectrum red emissions with EL peaks at 598 and 602 nm, FWHMs of 56 and 76 nm, and CIE coordinates of (0.612, 0.384) and (0.601, 0.397), respectively. Their EL spectra are consistent with the PL spectra of the doped films, indicating that the emission originates from the radiative decay of the singlet excitons of O-QA and S-QA. Owing to the HLCT characteristics, O-QA and S-QA have good EL efficiencies with EQE_max_ of 7.36% and 14.54%, respectively, indicative of their potential applications in OLEDs.

## 3. Conclusions

In summary, two new luminescent compounds O-QA and S-QA are developed by adding oxygen or sulfur atoms to the polycyclic carbonyl/nitrogen skeleton. They are thermally stable, and show PL peaks at 586 and 579 nm with narrow FWHMs of 48 and 64 nm in toluene solutions, and slightly red-shifted PL peaks at 598 and 600 nm with FWHMs of 55 and 79 nm in doped films. Theoretical calculations and experimental measurements reveal that both compounds have HLCT characteristics and the hRISC process may occur in both compounds, which facilitates the utilization of triplet excitons and thus is conducive to improving EL performance. At a doping concentration of 1 wt%, the device of O-QA attains the best EL performance, with an EQE_max_ of 7.36%, an EL peak at 598 nm, a FWHM of 56 nm and CIE coordinate of (0.612, 0.384). For S-QA, at a doping concentration of 5 wt%, a better EQE_max_ of 14.54% is obtained, with an EL peak at 602 nm, a FWHM of 76 nm and CIE coordinates of (0.601, 0.397). These results indicate that both new polycyclic luminescent compounds have good potential in OLEDs. Meanwhile, the comparison of EQE_max_ suggests that S-QA has better EL performance than O-QA, and the introduction of sulfur-containing fragments has higher potential for further exploration of red luminescent materials for OLED. The structure relationship gained in this work could be helpful for further exploration of red luminescent materials with narrow emission spectra.

## Figures and Tables

**Table 1:** Photophysical properties of O-QA and S-QA.

Compound	*λ*_abs_ ^(a)^ [nm]	*ε*_max_ ^(b)^ [10^4^]	*λ*_em_ ^(c)^ [nm]	FWHM ^(d)^ [nm]	Δ*E*_ST_ ^(e)^ [eV]	*Φ*_PL_ ^(f)^ [%]	*τ* ^(g)^ [ns]	*k*_r_ ^(h)^ [10^7^ s^−1^]
O-QA	550	3.15	586/598	48/55	0.29	55/67	18.6	3.60
S-QA	524	2.28	579/600	64/79	0.26	49/60	11.1	5.41

^(a)^ Absorption maximum (10^−5^ M); ^(b)^ Molar absorption coefficient corresponding to absorption maximum; ^(c)^ Emission peaks in toluene/doped film; ^(d)^ Full width at half maximum in toluene/doped film; ^(e)^ Singlet-triplet energy splitting in doped film; ^(f)^ Photoluminescence quantum yield in toluene/doped film; ^(g)^ Mean PL lifetime in doped films; ^(h)^ Radiative decay rate.

**Table 2:** EL performances of doped OLEDs based on O-QA and S-QA ^(a)^.

Emitter	*λ*_EL_ [nm]	*V*_on_ [V]	*L*_max_ [cd m^−2^]	CE_max_ [cd A^−1^]	PE_max_ [lm W^−1^]	EQE_max_ [%]	CIE (x, y)	FWHM [nm]
1 wt% O-QA	598	3.7	5439	13.58	11.53	7.36	(0.612, 0.384)	56
3 wt% O-QA	602	3.6	4601	11.18	9.7	6.70	(0.625, 0.373)	59
5 wt% O-QA	604	3.6	4039	8.70	7.59	5.46	(0.629, 0.369)	63
10 wt% O-QA	606	3.5	3494	5.71	5.13	3.80	(0.634, 0.364)	68
1 wt% S-QA	594	3.5	7784	18.96	17.01	8.76	(0.578, 0.417)	72
3 wt% S-QA	598	3.5	7392	26.20	23.52	13.27	(0.591, 0.405)	73
5 wt% S-QA	602	3.4	6482	26.62	24.60	14.54	(0.601, 0.397)	76
10 wt% S-QA	602	3.4	6483	13.89	12.84	8.02	(0.607, 0.391)	77

^(a)^ Abbreviations: *λ*_EL_ = EL peak; *V*_on_ = turn-on voltage at 1 cd m^−2^; *L*_max_ = maximum luminance; CE_max_ = maximum current efficiency; PE_max_ = maximum power efficiency; EQE_max_ = maximum external quantum efficiency; CIE = Commission Internationale de I’Eclairage coordinates; FWHM = full width at half maximum.

## Data Availability

The original contributions presented in this study are included in the article/Supplementary Material. Further inquiries can be directed to the corresponding author.

## Supplementary Materials

The following supporting information can be downloaded at: https://www.mdpi.com/article/10.3390/ma18174000/s1, Figure S1: ^1^H NMR spectrum of compound 1 in DMSO-*d*_6_; Figure S2: ^1^H NMR spectrum of compound 3 in DMSO-*d*_6_; Figure S3: ^1^H NMR spectrum of compound O-QA in C_2_D_2_Cl_4_. Figure S4: ^1^H NMR spectrum of compound 2 in DMSO-*d*_6_. Figure S5: ^1^H NMR spectrum of compound 4 in DMSO-*d*_6_. Figure S6: ^1^H NMR spectrum of compound S-QA in C_2_D_2_Cl_4_. Figure S7: (a) TGA thermograms of O-QA and S-QA, recorded under nitrogen at a heating rate of 10 °C min^−1^, *T*_d_ is decomposition temperature; (b) DSC thermograms of O-QA and S-QA, recorded under nitrogen at a heating rate of 10 °C min^−1^. (c) Cyclic voltammograms of O-QA and S-QA in dichloromethane. Figure S8: Comparison of optimized structures of O-QA and S-QA in S_0_ (pink) and S_1_ (blue) states. Figure S9: Natural transition orbital distributions of norm of highest occupied (HONTO) and lowest unoccupied natural transition orbital (LUNTO) of S_1_ state of O-QA and S-QA. Figure S10: (a) UV-vis absorption spectra and (b) PL spectra of O-QA, and (c) UV-vis absorption spectra and (d) PL spectra of S-QA in different solvents with varied polarity. Figure S11: Transient PL decay curves of O-QA and S-QA in toluene solutions. Figure S12: Fluorescence and phosphorescence spectra of O-QA (a) and S-QA (b) in doped films at 77 K. Figure S13: (a,b) EL spectra, (c,d) external quantum efficiency–luminance and (e,f) plots of luminance–voltage–current density of the OLEDs based on O-QA and S-QA doped in *m*CPBC host with different doping concentrations. Table S1: Absorption and emission peak positions of O-QA in different solvents. Table S2: Absorption and emission peak positions of S-QA in different solvents.
