# Supplementary material for: New Polycyclic Red Luminescent Compounds Based on Carbonyl/Nitrogen Skeleton for Efficient Narrow-Spectrum OLEDs
Source: Materials (Basel). 2025 Aug 26;18(17):4000. doi: 10.3390/ma18174000 (PMC12429404; doi:10.3390/ma18174000)
Supplement: Supplementary file 1 [file materials-18-04000-s001.zip › materials-3783672-supplementary.pdf]

materials

New Polycyclic Red Luminescent Compounds Based on Carbonyl/Nitrogen Skeleton for Efficient Narrow-Spectrum OLEDs

Zhiwei Wu [1], Peng Zou [1], Ziwei Chen [1], Ben Zhong Tang [2] and Zujin Zhao [1,*]

[1] State Key Laboratory of Luminescent Materials and Devices, Guangdong Provincial Key Laboratory of Luminescence from Molecular Aggregates, South China University of Technology, Guangzhou 510640, China; ziweichen980515@163.com (Z.C.)
[2] Guangdong Basic Research Center of Excellence for Aggregate Science, School of Science and Engineering, The Chinese University of Hong Kong, Shenzhen 518172, China
* Correspondence: mszjzhao@scut.edu.cn

S1. General information

The chemicals and reagents were purchased from commercial sources and used directly. The target products were obtained by silica-gel column chromatography and further purified via vacuum sublimation before photoluminescence (PL) and electroluminescence (EL) property examination. ^{1}H spectra were collected on a Bruker AV 400 spectrometer in DMSO-d_6 or $C_2D_2Cl_4$ solvent. High-resolution mass spectra (HRMS) were characterized on Agilent1290/Bruker maXis impact. Thermogravimetric analysis (TGA) was performed on Netzsch TG 209 under nitrogen flow at a heating rate of 10 °C min $^{-1}$. Differential scanning calorimetric (DSC) was performed on Netzsch DSC 200 F3 under nitrogen flow at a heating rate of 10 °C min $^{-1}$. UV-vis absorption spectra were tested on a Shimadzu UV-2600 spectrophotometer. PL spectra were recorded on a Horiba Fluoromax-4 spectrofluorometer. The temperature-dependent transient PL decay spectra were measured using FLS1000 fluorometer (Edinburgh Instruments). PL quantum yields (Φ_{PLS}) were obtained via a Hamamatsu absolute PL quantum yield spectrometer C11347 Quantaurus_QY. Cycle voltammetry (CV) curves were gained in dichloromethane using tetra-*n*-butylammonium hexafluorophosphate (Bu_4NPF_6, 0.1 M) as supporting electrolyte at a scan rate of 50 mV s^{-1}. Glassy carbon, platinum wire, and Ag/Ag^+ electrodes were used as work, counter, and reference electrodes, respectively. The highest occupied molecular orbital (HOMO) energy levels were obtained via the equation: $E_{HOMO} = -(E^{ox} - E^{ox}_{Fc/Fc+} + 4.8)$ eV, and the lowest unoccupied molecular orbital (LUMO) energy levels were calculated from the equation: $E_{LUMO} = E_{HOMO} + E_g$, (the E_g represents the optical energy gap). Based on density functional theory (DFT) and time-dependent density functional theory (TD-DFT), the ground-state and the first singlet excited-state geometry optimization and frequency analysis were conducted at PBE0/6-31G(d,p) level. The above-mentioned calculations were performed on the Gaussian 16 program package. The hole and electron analysis of singlet and triplet excited states were conducted with Multiwfn_3.8 code. The graphic rendering of molecular geometries and hole/electron distribution were obtained in VMD.

S2. OLED fabrication and characterization

Glass substrates pre-coated with a 90-nm-thin layer of indium tin oxide (ITO) with a sheet resistance of 20 Ω per square were thoroughly cleaned for 10 minutes in ultrasonic bath of acetone, isopropyl alcohol, detergent, deionized water, and isopropyl alcohol and then treated with O_2 plasma for 5 minutes in sequence. Organic layers were deposited onto the ITO-coated substrates by high-vacuum ($< 5 \times 10^{-4}$ Pa) thermal evaporation.

 https://doi.org/10.3390/ma18174000

Deposition rates were controlled by independent quartz crystal oscillators, which were 1~2 Å s^{-1} for organic materials, 0.2 Å s^{-1} for LiF, and 5 Å s^{-1} for Al, respectively. The emission area of the device was 3 × 3 mm^2 as shaped by the overlapping area of the anode and cathode. All the characterizations were conducted at room temperature in ambient atmosphere without any encapsulation and EL spectra were obtained via a PhotoResearch PR670 spectroradiometer, with a Keithley 2400 Source Meter. The external quantum efficiencies were estimated utilizing the normalized EL spectra and the current efficiencies of the devices, assuming that the devices were Lambertian emitters.

S3. Synthesis

S3.1. Synthesis of diethyl 2,5-di(10H-phenoxazin-10-yl)terephthalate (1)

A mixture of 2,5-dibromoterephthalic acid diethyl ester (1.90 g, 5.00 mmol), phenoxazine (2.01 g, 11.00 mmol), K_2CO_3 (2.76 g, 20.00 mmol), CuI (0.38 g, 2.00 mmol), and 18-crown-6 ether (0.53 g, 2.00 mmol) was added to a two-neck flask. The reaction was filled with nitrogen, and then dry 1,2-dichlorobenzene (50 mL) was injected into the flask. The mixture was heated to 180 °C and stirred for 24 h. After cooling to room temperature, the reaction mixture was poured into water, and extracted with dichloromethane three times. The combined organic layers were dried with anhydrous $MgSO_4$ and concentrated under reduced pressure after filtration. The crude product was further purified via silica-gel column chromatography using dichloromethane/petroleum ether as eluent to afford compound **1** as pale purple solid. Yield: 42%.^{1}H NMR (400 MHz, DMSO-d_6) δ (TMS, ppm): 8.17 (s, 2H), 6.78–6.70 (m, 12H), 6.06–6.04 (m, 4H), 4.12–4.06 (m, 4H), 1.01–0.98 (m, 6H).

S3.2. Synthesis of diethyl 2,5-di(10H-phenothiazin-10-yl)terephthalate (2)

Compound **2** was obtained by the analogous produces described for compound **1**. Yield: 40%. ^{1}H NMR (400 MHz, DMSO-d_6) δ (TMS, ppm): 8.19 (s, 2H), 7.08–7.06 (m, 4H), 6.99–6.95 (m, 4H), 6.89–6.86 (m, 4H), 6.20 (d, J = 6.4 Hz, 4H), 4.11–4.07 (m, 4H), 0.95–0.92 (m, 6H).

S3.3. Synthesis of diethyl 2,5-di(10H-phenoxazin-10-yl)terephthalate (3)

A round bottom flask was charged with compound **1** (2.93 g, 5.00 mmol), NaOH (2.00 g, 50.00 mmol) and THF/EtOH/H_2O (120 mL, 2:1:1, v/v/v). The reaction mixture was heated to reflux for 24 h, and then cooled down to room temperature. The mixture was concentrated via rotary evaporator under reduced pressure. The obtained solid was suspended in water (100 mL) and acidified with dilute hydrochloric acid. Compound **3** was collected as solid by vacuum filtration. Then, the solid was placed in a vacuum drying oven and dried at 80 °C for 12 h. This product was directly used in the subsequent reaction without further purification. Yield: 96%. ^{1}H NMR (400 MHz, DMSO-d_6) δ (TMS, ppm): 9.09 (s, 2H), 6.75–6.69 (m, 12H), 6.03–6.00 (m, 4H).

S3.4. Synthesis of 2,5-di(10H-phenothiazin-10-yl)terephthalic acid (4)

Compound **4** was obtained by the analogous produces described for compound **3**. Yield: 96%. ^{1}H NMR (400 MHz, DMSO-d_6) δ (TMS, ppm): 13.52 (s, 2H), 8.09 (s, 2H), 7.04–7.02 (m, 4H), 6.97–6.94 (m, 4H), 6.86–6.83 (m, 4H), 6.17–6.15 (m, 4H).

S3.5. Synthesis of compound O-QA

Compound **2** (2.64 g, 5.00 mmol) was added to a two-neck flask, which was filled with nitrogen, and then dry dichloromethane (40 mL), oxalyl chloride (2.54 g, 20.00 mmol), and *N,N*-dimethylformamide (0.05 mL) were injected into the flask. The reaction mixture was heated to reflux and stirred for 3 h, then was added with $SnCl_4$ (5.22 g, 20.00 mmol)

and stirred for further 4 h. The reaction mixture was quenched with NaOH aqueous solution and the generated red solid product O-QA was collected through filtration. Yield: 82%. ^{1}H NMR (400 MHz, $C_2D_2Cl_4$) δ (TMS, ppm): 9.21 (s, 2H), 8.01–7.98 (m, 2H), 7.80 (d, *J* = 7.6 Hz, 2H), 7.37–7.23 (m, 10H). HRMS ($C_{32}H_{17}N_2O_4$): *m*/*z* [M + H$^+$] calcd 493.1183, found 493.1185.

S3.6. Synthesis of compound S-QA

S-QA was obtained by the analogous produces described for O-QA. Red solid product (Yield: 80%). ^{1}H NMR (400 MHz, $C_2D_2Cl_4$) δ (TMS, ppm): 8.94 (s, 2H), 8.21–8.19 (m, 2H), 7.69–7.30 (m, 12H). HRMS ($C_{32}H_{17}N_2O_2S_2$): *m*/*z* [M + H$^+$] calcd 525.0726, found 525.0732.

S4. Supported data

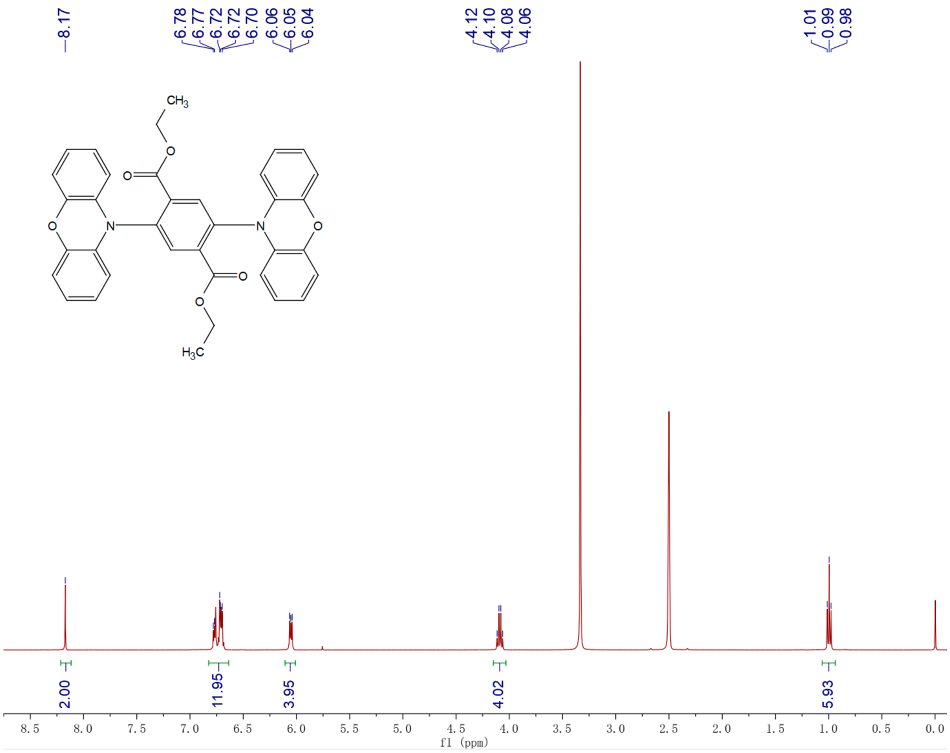

Figure S1. ^{1}H NMR spectrum of compound **1** in DMSO-d_6.

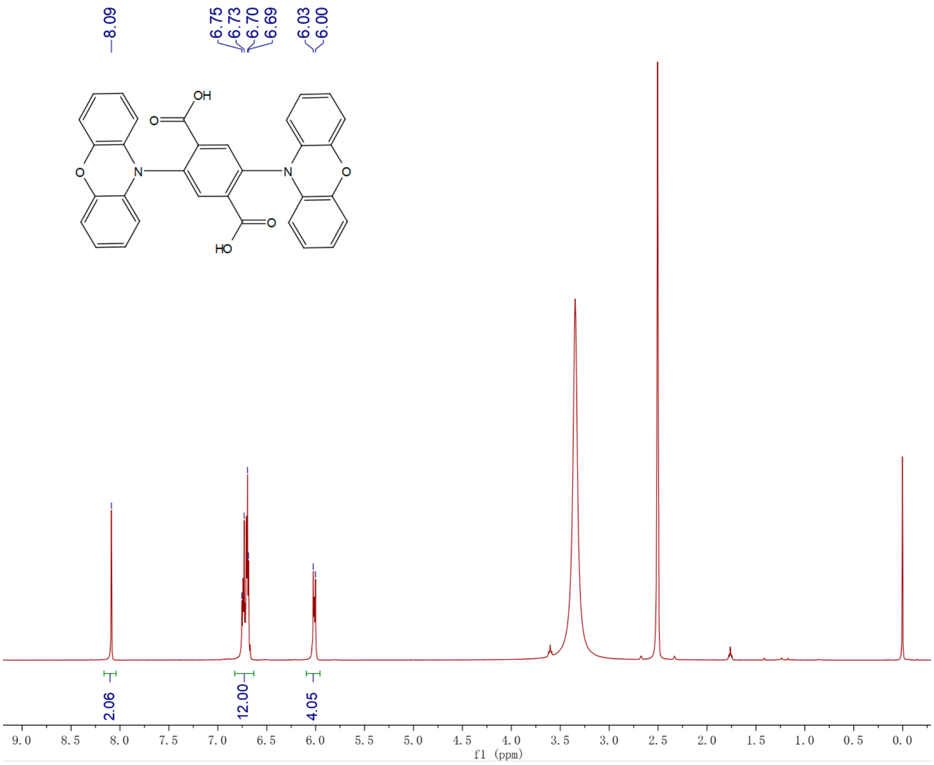

Figure S2. ^{1}H NMR spectrum of compound **3** in DMSO-*d*6.

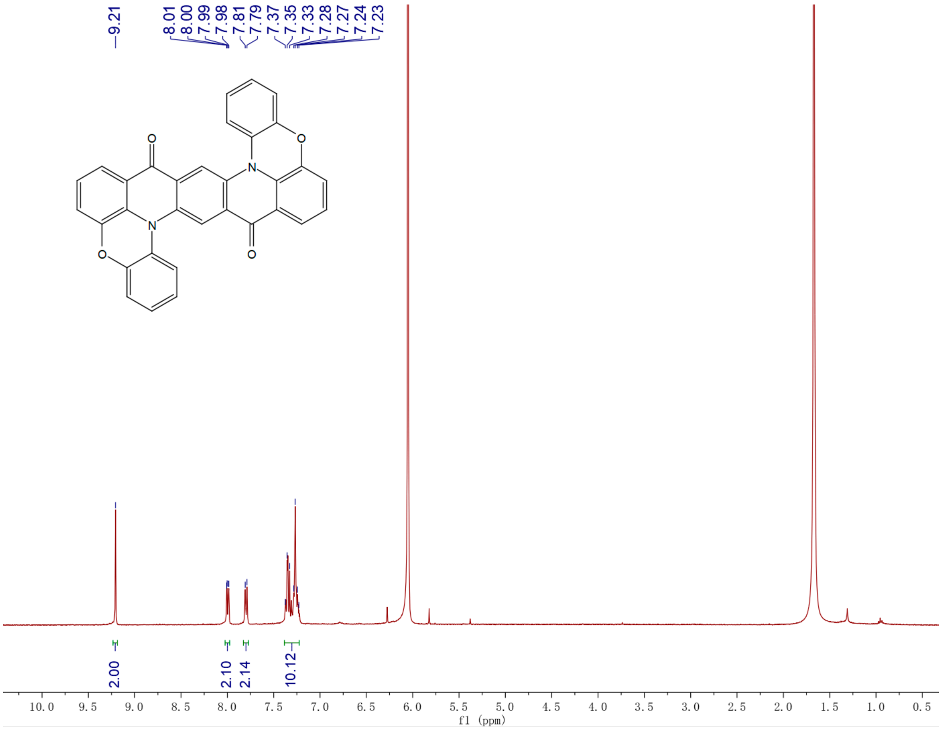

Figure S3. ^{1}H NMR spectrum of compound O-QA in $C_2D_2Cl_4$.

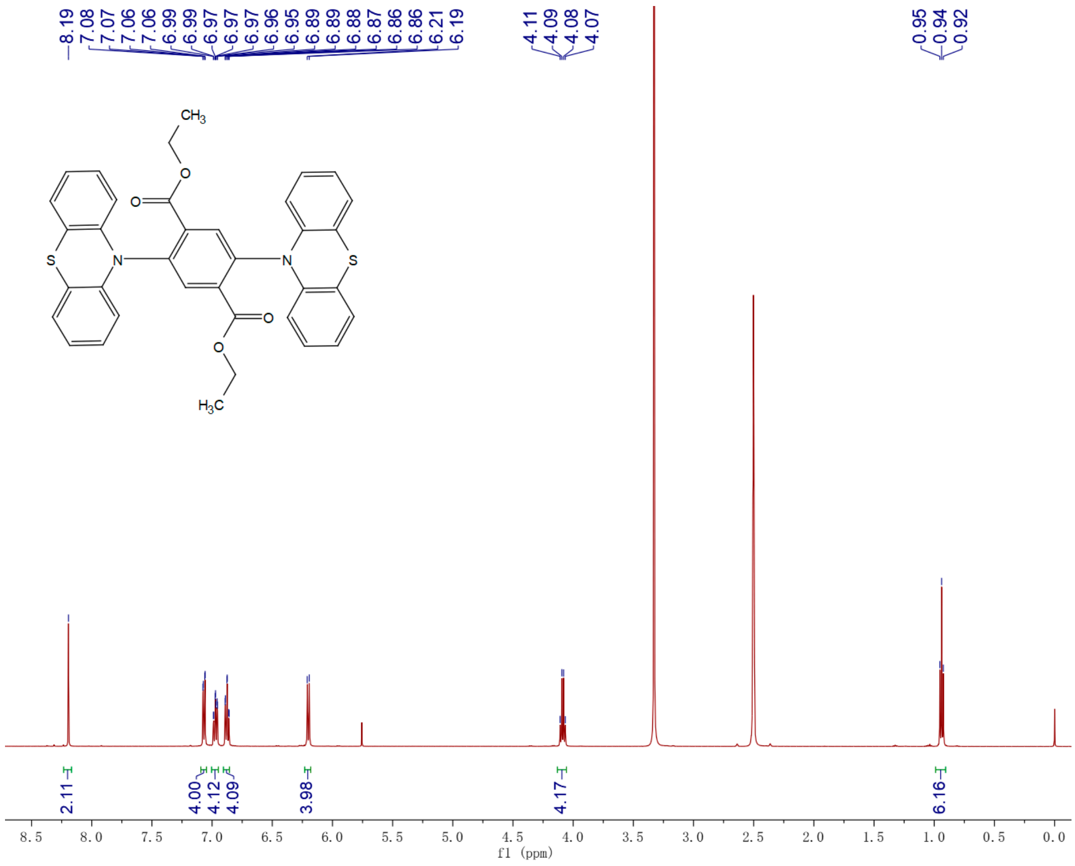

Figure S4. ^{1}H NMR spectrum of compound **2** in DMSO-d_6.

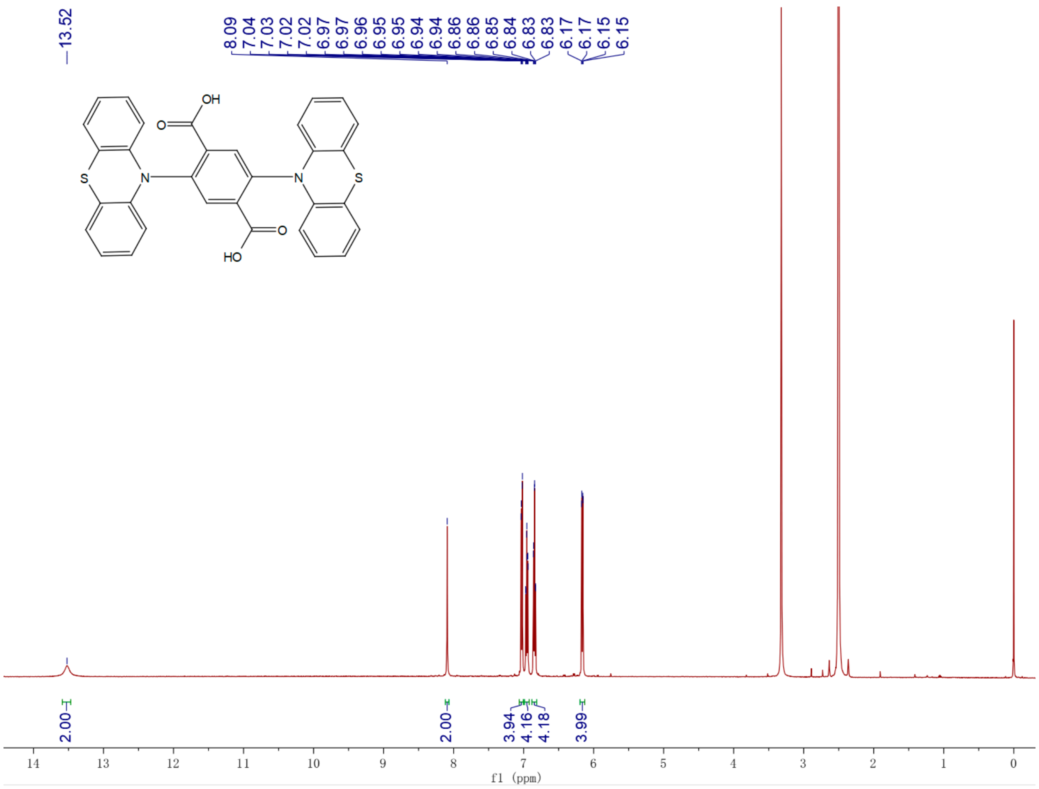

Figure S5. ^{1}H NMR spectrum of compound **4** in DMSO-d_6.

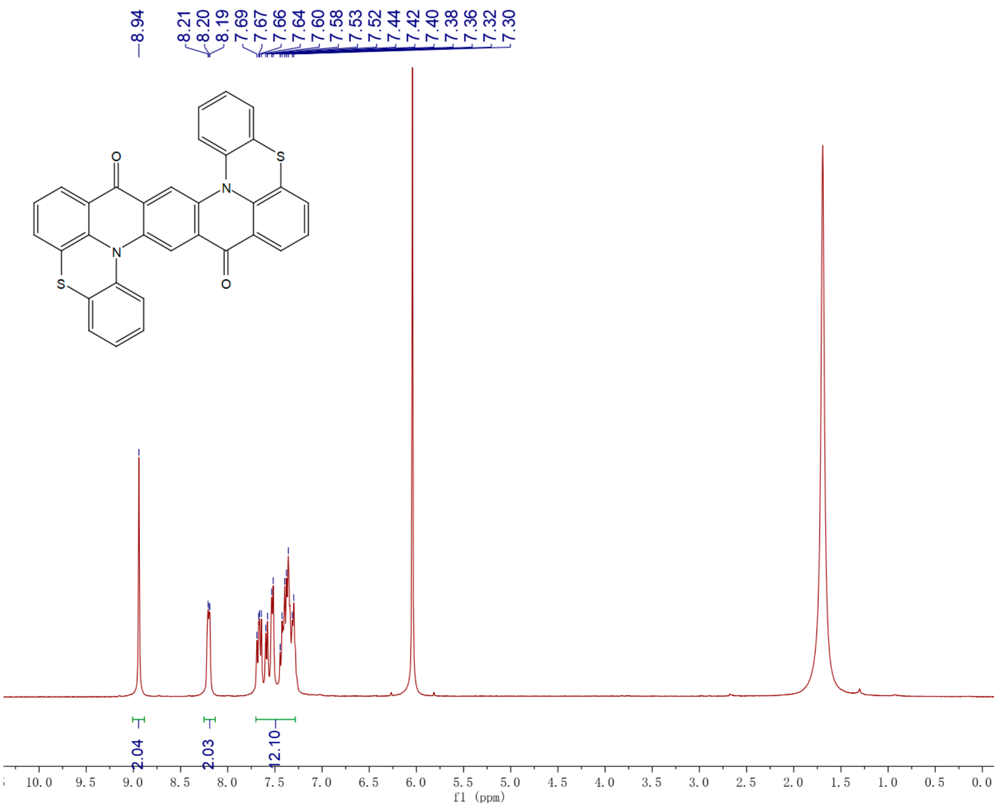

Figure S6. ^{1}H NMR spectrum of compound S-QA in $C_2D_2Cl_4$.

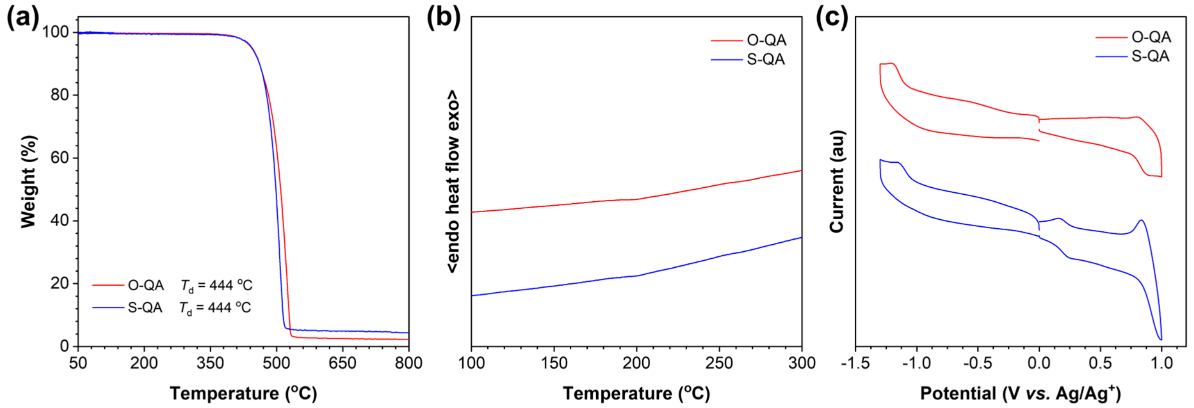

Figure S7. (a) TGA thermograms of O-QA and S-QA, recorded under nitrogen at a heating rate of 10 °C min^{-1}, T_d is decomposition temperature; (b) DSC thermograms of O-QA and S-QA, recorded under nitrogen at a heating rate of 10 °C min^{-1}; (c) cyclic voltammograms of O-QA and S-QA in dichloromethane.

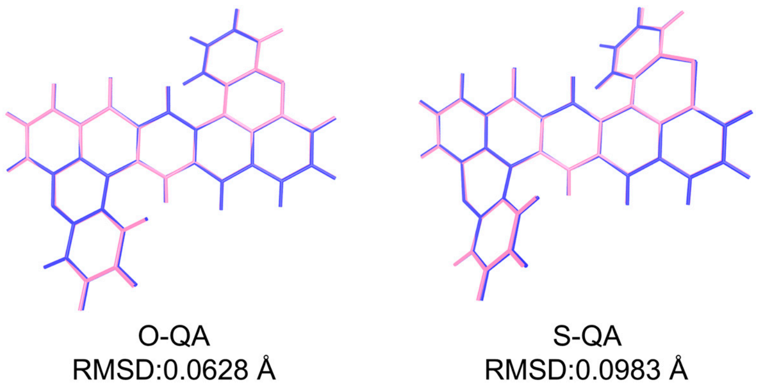

Figure S8. Comparison of optimized structures of O-QA and S-QA in S_0 (pink) and S_1 (blue) states.

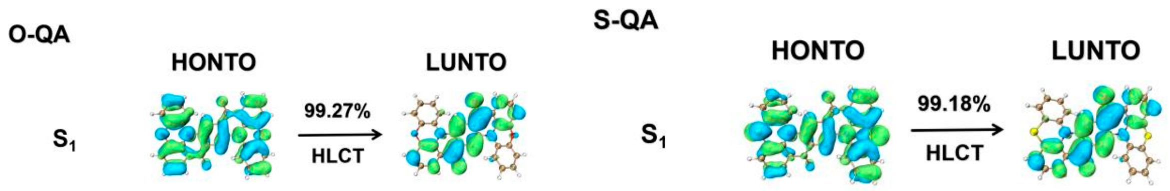

Figure S9. Natural transition orbital distributions of the norm of highest occupied (HONTO) and lowest unoccupied natural transition orbital (LUNTO) of S1 state of O-QA and S-QA.

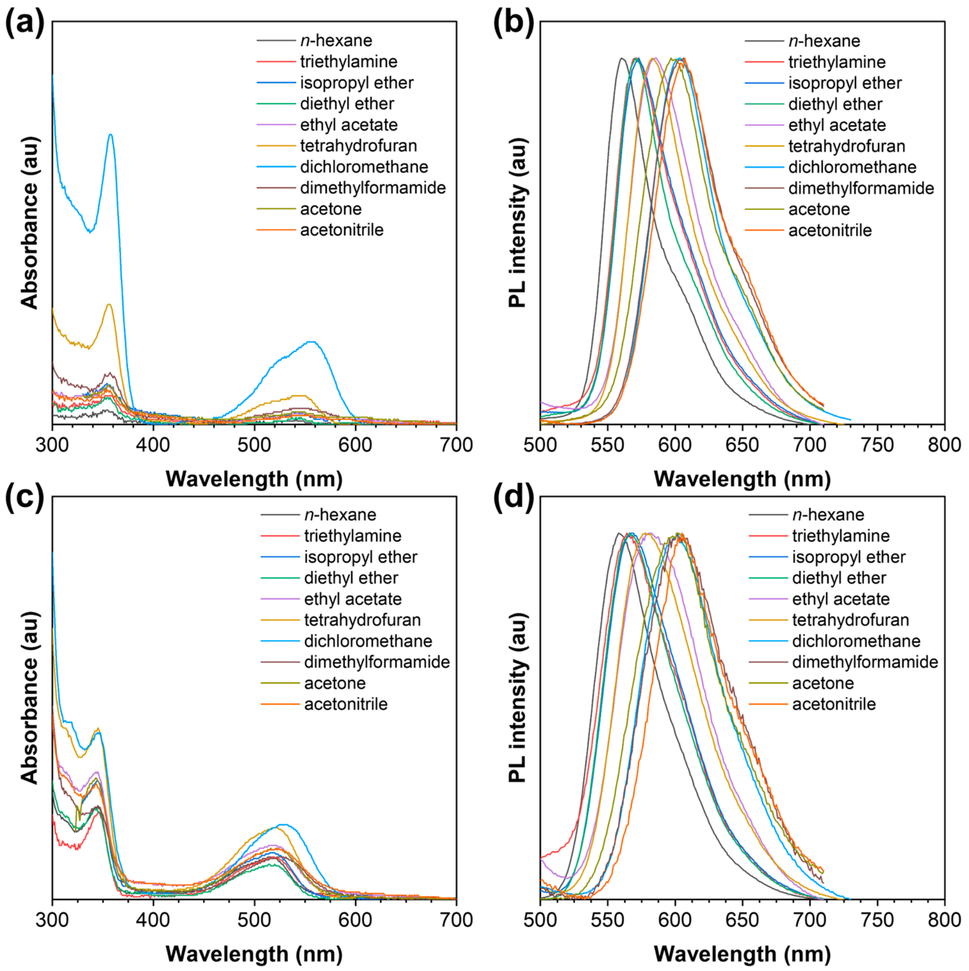

Figure S10. (a) UV-vis absorption spectra and (b) PL spectra of O-QA, and (c) UV-vis absorption spectra and (d) PL spectra of S-QA in different solvents with varied polarity.

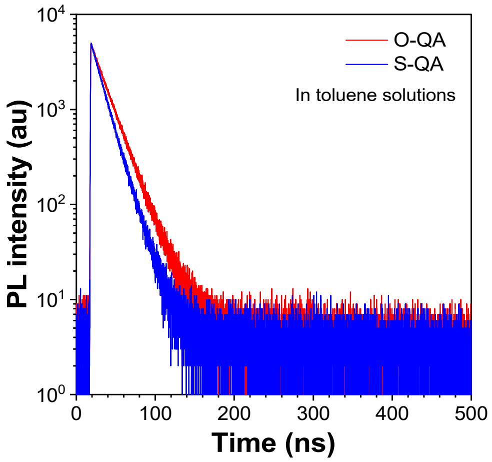

Figure S11. Transient PL decay curves of O-QA and S-QA in toluene solutions.

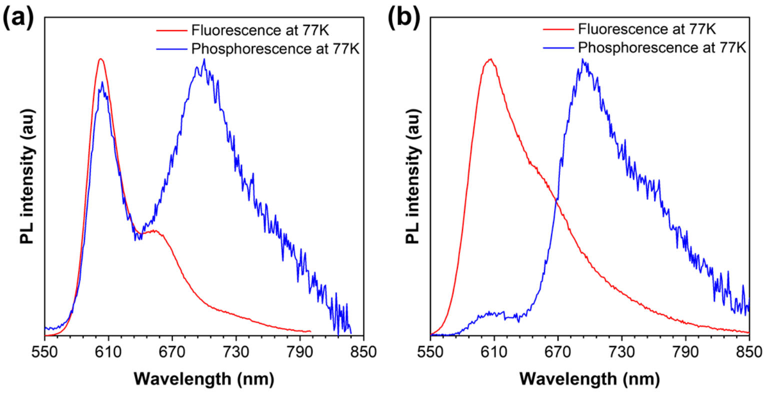

Figure S12. Fluorescence and phosphorescence spectra of O-QA (a) and S-QA (b) in doped films at 77K.

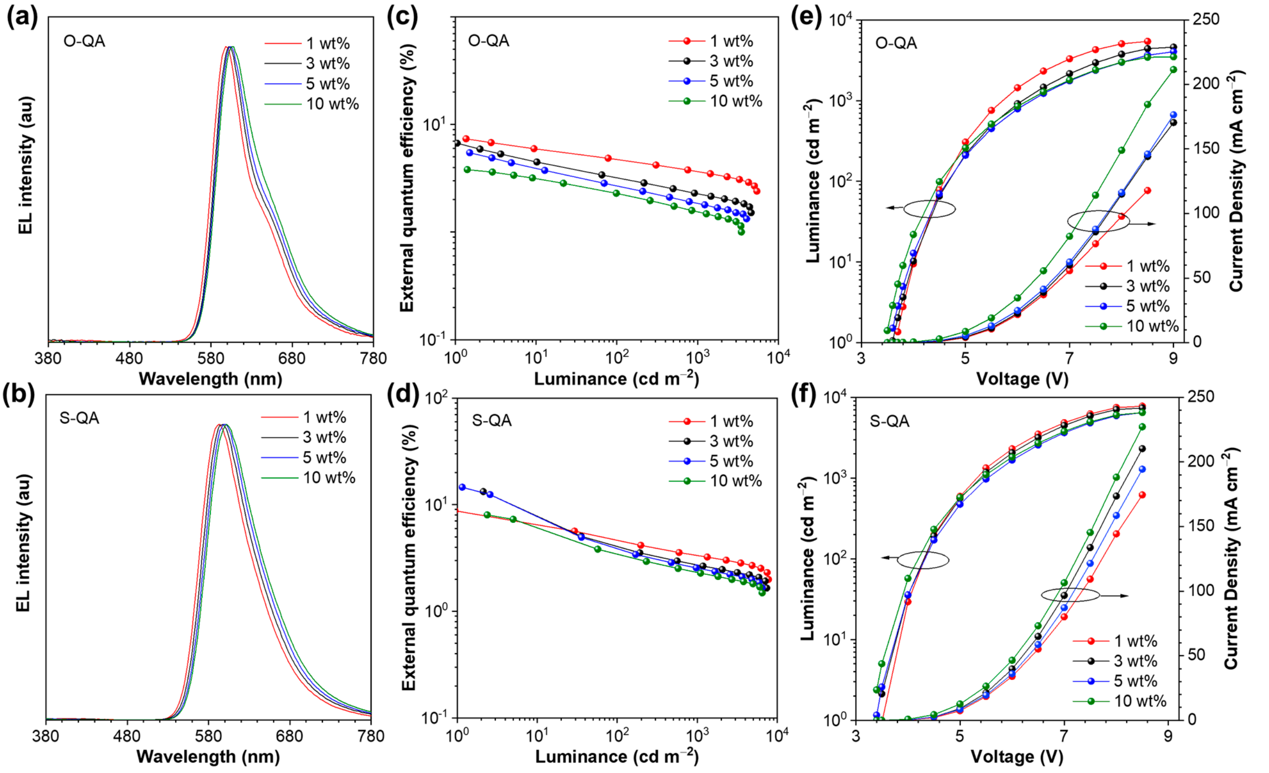

Figure S13. (a,b) EL spectra, (c,d) external quantum efficiency–luminance and (e,f) plots of luminance–voltage–current density of the OLEDs based on O-QA and S-QA doped in *m*CPBC host with different doping concentrations.

Table S1. Absorption and emission peak positions of O-QA in different solvents.

solvent	ε	n	f [ε,n]	λ_{abs} [nm]	λ_{em} [nm]	$v_a - v_f$
hexane	1.9	1.375	0.0012	541	560	627
triethylamine	2.42	1.401	0.048	544	571	869
isopropyl ether	3.88	1.368	0.145	540	573	1067
diethyl ether	4.34	1.352	0.167	541	570	940
ethyl acetate	6.02	1.372	0.200	545	586	1284
tetrahydrofuran	7.58	1.407	0.210	543	583	1264
dichloromethane	8.93	1.424	0.217	556	603	1402
dimethylformamide	37	1.427	0.276	556	606	1484
acetone	20.7	1.359	0.284	544	597	1632
acetonitrile	37.5	1.344	0.305	547	607	1807

Table S2. Absorption and emission peak positions of S-QA in different solvents.

solvent	ε	n	f [ε,n]	λ_{abs} [nm]	λ_{em} [nm]	$v_a - v_f$
hexane	1.9	1.375	0.0012	515	558	1496
triethylamine	2.42	1.401	0.048	518	564	1575
isopropyl ether	3.88	1.368	0.145	517	568	1737
diethyl ether	4.34	1.352	0.167	515	566	1750
ethyl acetate	6.02	1.372	0.200	517	582	2160
tetrahydrofuran	7.58	1.407	0.210	520	578	1930
dichloromethane	8.93	1.424	0.217	529	601	2265

dimethylformamide	37	1.427	0.276	525	601	2409
acetone	20.7	1.359	0.284	522	602	2546
acetonitrile	37.5	1.344	0.305	521	605	2665
